# Effects of Nitrogen Fertilizers on the Growth and Nitrate Content of Lettuce (*Lactuca sativa* L.)

**DOI:** 10.3390/ijerph110404427

**Published:** 2014-04-22

**Authors:** Cheng-Wei Liu, Yu Sung, Bo-Ching Chen, Hung-Yu Lai

**Affiliations:** 1 Department of Post-Modern Agriculture, MingDao University, Changhua 52345, Taiwan; E-Mails: chad888@mdu.edu.tw (C.-W.L.), bcchen@mdu.edu.tw (B.-C.C.); 2 Department of Horticulture, National Chung Hsing University, Taichung 40227, Taiwan; E-Mail: yusung@dragon.nchu.edu.tw

**Keywords:** inorganic fertilizer, lettuce, liquid fertilizer, nitrate, organic fertilizer

## Abstract

Nitrogen is an essential element for plant growth and development; however, due to environmental pollution, high nitrate concentrations accumulate in the edible parts of these leafy vegetables, particularly if excessive nitrogen fertilizer has been applied. Consuming these crops can harm human health; thus, developing a suitable strategy for the agricultural application of nitrogen fertilizer is important. Organic, inorganic, and liquid fertilizers were utilized in this study to investigate their effect on nitrate concentrations and lettuce growth. The results of this pot experiment show that the total nitrogen concentration in soil and the nitrate concentration in lettuce increased as the amount of nitrogen fertilizer increased. If the recommended amount of inorganic fertilizer (200 kg·N·ha^−1^) is used as a standard of comparison, lettuce augmented with organic fertilizers (200 kg·N·ha^−1^) have significantly longer and wider leaves, higher shoot, and lower concentrations of nitrate.

## 1. Introduction

Nitrogen is an essential element required for successful plant growth. Although inorganic nitrogen compounds (*i.e.*, NH_4_^+^, NO_2_^‒^, and NO_3_^‒^) account for less than 5% of the total nitrogen in soil [1], they are the main form of the element absorbed by most plants. Inorganic and organic fertilizers are applied to maintain the nutritional condition of different cropping systems. For an organic agricultural system, continuous application of manure increases the nitrogen (N), phosphorus (P), potassium (K), calcium, and magnesium content in soil [2,3]. Once organic fertilizers are applied to soils and mineralization begins, inorganic nitrogen is released and absorbed by plants. However, the rate of mineralization is controlled by several factors, including agricultural management, microorganism, soil properties, temperature, and water content [4,5,6], as well as the type of organic fertilizer [7]. Many models have been developed to predict the release of nitrogen in applied organic fertilizers [6,8].

Once nitrogen fertilizers are applied to agricultural systems, the fertilizers are absorbed directly by plants or converted into various other forms through the oxidation process. Excess nitrogen is lost in ionic or gaseous form through leaching, volatilization, and denitrification [1,9]. If nitrate is not absorbed by plant roots, it is carried away by runoff or leaches into the soil along with water [9]. The phytoavailability of the nitrogen pool increases when excess nitrogen is applied, and this increase intensifies the potential threat to the surrounding environment [10]. There are close relationships between the excessive application of nitrogen fertilizers and environmental problems such as eutrophication, the greenhouse effect, and acid rain [11,12]. Consuming contaminated groundwater or crops with a high concentration of nitrate has negative effects on human health [13]. In a study by Donner and Kucharik [14], when the application rate of nitrogen fertilizer was increased by 30%, the corn yield increased 4%, but the amount of nitrate lost through leaching increased by 53%. Although the yield decreased by 10% when the application rate of nitrogen fertilizer was reduced by 30%, the leaching loss was 37% less. Applying manure (150 kg·N·ha^−1^·year^−1^) raises the yield of *Phleum pratense* L. Champ; however, the excess nitrogen that accumulates in the soil if double the amount of manure is applied may result in a decrease in yield [10]. According to the results of previous studies, the accumulation of nitrates in the edible parts of crops is directly related to the type of nitrogen fertilizer used [15,16], as well as the soil properties. Regarding lettuce, the light intensity [17], timing of fertilizer-N release [18], and lettuce type [19] have been shown to affect the accumulation of nitrates in this crop.

Rationalizing fertilizer application is an important issue for sustainable agriculture because it can reduce the negative effects of farming on the surrounding environment [20]. An agricultural system should include yield and environmental quality during management. Green leafy vegetables contain the highest nitrate levels [21], and lettuce is classified as having very high nitrate content [22]. Because consuming high levels of nitrate may further lead to severe pathologies in humans [23], cultivating edible crops with low nitrate content is very important. The Joint Expert Committee of the Food and Agriculture (JECFA) Organization of the United Nations/World Health Organization and the European Commission (EC) Scientific Committee on Food have also set an acceptable daily intake for nitrate of 0–3.7 mg·kg^−1^ body weight [22]. The U.S. Environmental Protection Agency (EPA) reference dose for nitrate is equivalent to about 7.0 mg·kg^−1^ body weight per day [23].

In this study, a pot experiment was conducted using various types of nitrogen fertilizers, application rates, and combinations of rates and fertilizers. The objective of the present study is to develop an agricultural system that produces healthy lettuce that has low nitrate content.

## 2. Experimental Section

### 2.1. Soil Sampling and Analysis

Surface soil samples (0–15 cm) were collected from the organic farm at MingDao University. After the visible pieces of gravel and debris were removed, the samples were air-dried, ground, and passed through a 5-mesh stainless steel sieve in order to obtain soil samples for the pot experiment. Some soil samples were ground further and passed through 10-mesh or 80-mesh stainless steel sieves for physical and chemical analysis. Selected properties were analyzed, including water content [24], pH (w/v = 1/1) [25], electrical conductivity (EC; w/v = 1/1) [26], texture [27], soil organic carbon (SOC) [28], cation exchange capacity (CEC) [29], and total Kjeldahl nitrogen (TKN) [30].

### 2.2. Organic and Liquid Fertilizers

A commercial organic fertilizer (Code 0687009, Sinon Corporation, Taichung, Taiwan) was used in this study. Liquid fertilizer was prepared using tap water, powdered soy beans, rice bran, powdered seaweed, molasses, and commercial microorganisms. These materials were combined in a plastic container and stirred for approximately 5 min twice per day for 14 days. After 30 days of fermenting, the total nitrogen concentration in the diluted liquid fertilizer (25-fold) was determined and used in the pot experiment.

### 2.3. Pot Experiment

The amount of nitrogen fertilizer used in this study was based on the Council of Agriculture of Taiwan’s recommended amount (200 kg·N·ha^−1^) for leafy vegetables. Only a two-fold amount of nitrogen fertilizer was applied as another treatment because the average amount applied by Taiwanese farmers to vegetables was approximately 250 kg·N·ha^−1^ [31]. In addition to nitrogen fertilizers, phosphate and potassium were included in this study in accordance with the fertilizer amounts recommended by the Council of Agriculture of Taiwan (P_2_O_5_-K_2_O = 100-100 kg·ha^−1^). Seven nitrogen fertilization treatments were compared with three replicates, including the following:

- CK: no fertilizers applied
- CF200: amended with 200 kg·N·ha^−1^ as NH_4_NO_3_
- CF400: amended with 400 kg·N·ha^−1^ as NH_4_NO_3_
- OM200: amended with 200 kg·N·ha^−1^ as organic fertilizer
- OM400: amended with 400 kg·N·ha^−1^ as organic fertilizer
- OM200+LF: in addition to organic fertilizer (200 kg·N·ha^−1^), 360 mL of diluted liquid fertilizer was applied every 3–4 days
- OM400+LF: in addition to organic fertilizer (400 kg·N·ha^−1^), 360 mL of diluted liquid fertilizer was applied every 3–4 days

The pot experiment was conducted for 50 days in a 25 °C phytotron (110 mol·sec^−1^·m^−2^; day/night = 12/12 h) at MingDao University. A mixture of soil and different types of fertilizer (4.5 kg·pot^−1^) was added to a square pot. The water content was set at 70% of the water-holding capacity (WHC) by adding deionized (DI) water, and 300 leaf lettuce seeds (*Lactuca sativa* L.) were sowed into the surface soil. The water content of the soil was controlled at 50%–70% of the WHC during the pot experiment by weighing the pot every 2–3 days.

### 2.4. Sampling and Analysis

Ten days after germination, the soil and edible parts of the lettuce were sampled simultaneously every 5–10 days. To get sufficient amounts of plants for determination and analysis, different numbers of seedlings were sampled at different growth periods. During days 10–29, days 30–39, and days 40–50 after germination, 30, 20, and 10 lettuce seedlings were sampled randomly, respectively. The shoot height (*H*), length (*L*), and width (*W*) were measured and recorded every 5–10 days. After the lettuce had grown for 50 days, the chlorophyll content of the biggest leaf was also measured and recorded using a Konica Minolta SPAD-502 (Konica Minolta, Tokyo, Japan). The tissues of the harvested lettuce were washed with tap water and DI water and oven dried at 65 °C for 72 h, and then the dry weight (*DW*) was determined. The dry tissues were further ground using a grinder and then digested using H_2_SO_4_/C_6_H_4_(OH)(COOH)/H_2_O_2_ [32]. The soil samples were air-dried, ground, passed through a 10-mesh stainless steel sieve, and then digested using H_2_SO_4_/C_6_H_4_(OH)(COOH) [30]. The total nitrogen concentration in the filtrate of the plants and soils passed through filter papers (Whatman No. 42, Kent, UK) was determined with an automatic distillation unit (Büchi K-350, Postfach, Switzerland). A statistical analysis was conducted using analysis of variance for the main effects; the means of the values were compared with the least significant difference (*p* = 0.05).

## 3. Results and Discussion

### 3.1. Basic Properties of Soil and Fertilizers

The pH of the tested soil was 7.72, and the EC (w/v = 1/1) was 0.19 dS·m^−1^. The soil had a moderate organic carbon content (1.21%) and a coarse texture (sandy loam). The non-diluted liquid fertilizer was extremely acidic (pH = 4.01) and had a high EC (18.4 dS·m^−1^). The total copper (Cu), zinc (Zn), N, P, and K concentrations were 15.5, 2.32, 466, 0.87, and 1070 mg·L^−1^, respectively. The organic fertilizer had mild alkalinity (pH = 7.81; w/v = 1/3), but the EC reached 12.2 dS·m^−1^ (w/v = 1/3). The total Cu, Zn, N, P, and K concentrations were 110, 209, 39,599, 337, and 19,583 mg·kg^−1^, respectively.

### 3.2. Effects on Soil Properties

The addition to the various fertilizers influenced or significantly influenced (*p* < 0.05) the EC of the potted soils collected at the end of the experiment (Table 1). Relative to the CK soil, the application of various fertilizers raised the EC of the soils to different levels, and the soil treated with CF400 had the highest EC value (1.21 dS·m^−1^) of all the treatments. Although the EC values were lower than the threshold of a saline soil, *i.e.*, EC (w/v = 1/1) = 2.4 dS·m^−1^ [33], the applied fertilizers amounts should be controlled to avoid additional salt accumulation. The treatment with OM200 + LF and OM400 + LF increased the EC of the soil from 0.22 dS·m^−1^ (CK) to approximately 0.75 dS·m^−1^. Higher EC values could potentially decrease the water potential of the soil water and thus inhibit plant growth [34]. The organic fertilizer and liquid fertilizer have higher EC values, which may increase soil EC and thus affect plant growth after the fertilizers are applied to the land [35].

**Table 1:** Effects of different nitrogen fertilizer treatments on soil properties.

Treatments ^†^	Soil Properties ^‡^			
	pH_H_2_O_	EC dS·m^−1^	SOC %	TKN mg·kg^−1^
CK	7.83 (7.75–7.89) ^¶^ b	0.22 ± 0.04 ^§^ b	0.88 ± 0.02 ^§^ b	1,117 ± 156 ^§^ b
CF200	7.47 (7.32–7.60) c	0.64 ± 0.13 b	0.87 ± 0.03 b	1,337 ± 152 a
CF400	7.44 (7.32–7.65) c	1.21 ± 0.66 a	0.86 ± 0.01 b	1,249 ± 151 a
OM200	7.59 (7.40–7.75) bc	0.35 ± 0.07 b	0.87 ± 0.02 b	1,328 ± 197 a
OM400	7.61 (7.56–7.67) bc	0.56 ± 0.20 b	0.88 ± 0.03 b	1,300 ± 143 a
OM200 + LF	8.14 (8.09–8.20) a	0.75 ± 0.28 ab	0.98 ± 0.02 a	1,378 ± 173 a
OM400 + LF	8.25 (8.11–8.47) a	0.76 ± 0.07 ab	0.99 ± 0.05 a	1,434 ± 214 a

Notes: ^†^ CK—without applying any fertilizers; CF200—200 kg·N·ha^−1^ as NH_4_NO_3_; CF400—400 kg·N·ha^−1^ as NH_4_NO_3_; OM200—200 kg·N·ha^−1^ as organic fertilizer; OM400—400 kg·N·ha^−1^ as organic fertilizer; OM200 + LF—in addition to organic fertilizer (200 kg·N·ha^−1^), 360 mL of diluted liquid fertilizer was applied every 3–4 days; OM400 + LF—in addition to organic fertilizer (400 kg·N·ha^−1^), 360 mL of diluted liquid fertilizer was applied every 3–4 days; ^‡^ pH_H2O_ (w/v = 1/1); EC: Electrical conductivity (w/v = 1/1); SOC: soil organic carbon; TKN: Total Kjeldahl nitrogen. Values followed by different letters are statistically different (*p* < 0.05). ^¶^ mean (data range); ^§^ mean ± standard deviation (n = 3).

Because NH_4_NO_3_ was used in this study, the nitrification of NH_4_^+^ released H^+^, and the inorganic fertilizers significantly decreased the pH_H2O_ of the soil from 7.83 to 7.44–7.47 (*p* < 0.05). The pH of the non-diluted liquid fertilizer was 4.01, and changed to 6.65 after the fertilizer was diluted with DI water when the mixture was applied to the soil. Relative to the OM200 and OM400 treatments, the soils treated with the combination of organic fertilizer with liquid fertilizer had higher pH. This phenomenon possibly resulted from the high EC of the liquid fertilizer and thus increased the nonacid cation concentrations, which depleted the soil acidity [1]. However, it is not easy from the current data to clarify the role of liquid fertilizer because it may have interacted with the organic fertilizer. Possibly as a result of the lower application rate of the organic fertilizer (approximately 0.25%), for most of the treatments there was no significant effect of the different treatments on the SOC content, which was 0.88%–0.99% (Table 1). Compared with the CK soil, the increases in the total soil nitrogen in the various fertilizer treatments reached 10–30% (*p* < 0.05).

In Taiwan, the amount of nitrogen fertilizer recommended by the Council of Agriculture for leafy vegetables is 200 kg·N·ha^−1^. Our experimental results show that applying fertilizers significantly increases the TKN in soils (*p* < 0.05), regardless of fertilizer type. The difference in TKN in soil between treatment and after amending with different types of fertilizers was less than 8%. Without the application of fertilizers, *i.e.*, CK soil, the TKN in the soil was 17% less than the soil treated with CF200.

### 3.3. Effects on the Growth of Lettuce

**Figure 1:**
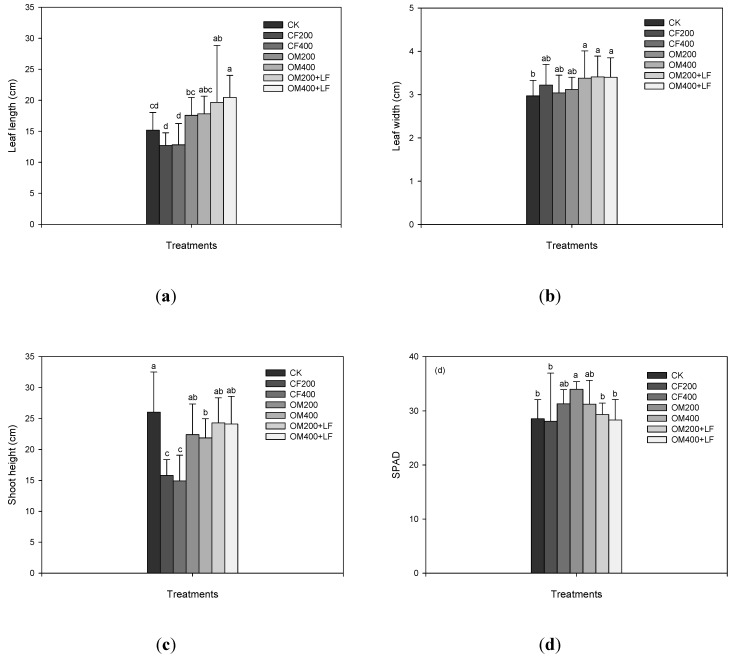
Effects of different treatments of nitrogen fertilizers on the (**a**) leaf length, (**b**) leaf width, (**c**) shoot height, and (**d**) SPAD of lettuce after 50 days of cultivation. Values followed by different letters in columns are statistically different (*p* < 0.05).

Compared with the CK-grown lettuce, the lettuce grown in soil amended with inorganic fertilizers had shorter (15%–17%; Figure 1a) and wider leaves (2%–9%; Figure 1b) while the treatments with organic fertilizers increased the length (15%–18%) and width (5%–14%) of the largest leaves although the differences were not significant. The combination of organic fertilizers with liquid fertilizers promoted the lettuce growth the most, and the increases in length and width reached 29%–35% and 14%–15%, respectively (*p* < 0.05). If the length and width of the leaves were used to estimate the leaf area (*LA*) of lettuce, *i.e.*, *LW*/2, compared with the CK lettuce, the increase in the *LA* in the lettuce treated with organic fertilizers combined with liquid fertilizers reached 48–55%. In the soil treated with inorganic fertilizers, however, the *LA* decreased (7%–15%) but was not statistically different compared with CK. Because the soil EC increased in the inorganic fertilizer treatments (Table 1), the inhibited CF200 and CF400 on the *L* and *H* possibly resulted from the change in soil EC. However, although the soil EC in the combination of organic fertilizers with liquid fertilizers was higher than in the inorganic fertilizer treatments (Table 1), the *L* and *H* were still higher compared with the CF200 and CF400 treatments. The effect may result from the higher soil pH values in the OM200 + LF and OM400 + LF treatments compared with OM200 and OM400. The concentrations of different cations possibly complex with carbonates in the alkaline environment [36], and thus decreased the availability and alleviated the negative effect of soil EC on the lettuce growth. Because only five soil properties were analyzed in this study, and the interaction between liquid and organic fertilizer was not clarified from the current data, soil properties other than EC may have affected the experimental result. All changes decreased or significantly decreased (*p* < 0.05) the shoot height of the lettuce compared to the CK lettuce (Figure 1c), and this was particularly the case with inorganic fertilizers (decreased 39%–43%). For most treatments (compared to the CK lettuce), the organic fertilizers or the combination of organic fertilizers with liquid fertilizers increased or significantly increased (*p* < 0.05) the SPAD values (2%–20%; Figure 1d).

**Figure 2:**
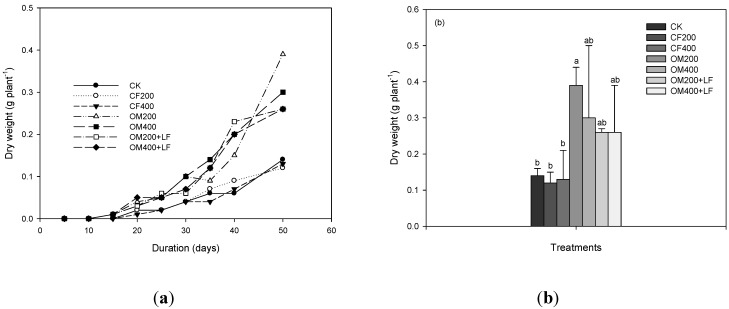
The (**a**) dynamic change in the dry weight and (**b**) dry weight at day 50 of lettuce grown in soils treated with different nitrogen fertilizers. Values followed by different letters in columns are statistically different (*p* < 0.05).

The differences in the *DW* were generally analogous to the differences between the leaf length and the shoot height (Figure 2). The *DW* of the lettuce increased with time (Figure 2a). After the lettuce had grown for 50 days, compared to the CK-grown lettuce, the *DW* of the lettuce grown in inorganic fertilizer-amended soils did not change significantly (Figure 2b). Changes with organic fertilizers or the combination of organic fertilizers with liquid fertilizers promoted or significantly promoted (*p* < 0.05) lettuce growth, and the *DW* increased 85%–180%.

**Figure 3:**
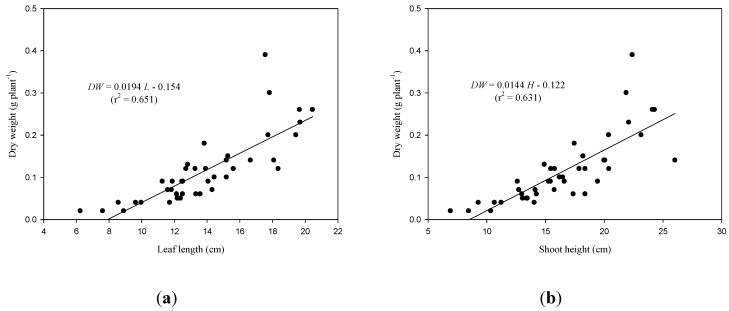
Linear relationships between growth parameters: (**a**) dry weight *vs.* leaf length and (**b**) dry eight *vs.* shoot height.

Treatment with organic fertilizers deepened the color of the lettuce leaves, and the SPAD measurement increased 11%–22% compared to the CK-treated plants. Relative to the CF200-treated lettuce, the organic fertilizers and the combination of organic fertilizers and liquid fertilizers promoted lettuce growth, especially the shoot height and the leaf length of the largest leaves (38%–61%). The estimated *LA* of the plants treated with organic fertilizers and the fertilizers that had an additional application of liquid fertilizer increased by between 33% and 77% compared to the CF200-treated lettuce, and the *DW* consequently increased 115%–150%. In a study, Pavlou *et al.* [16] reported that leaf width is strongly correlated to lettuce yield, and leaf length is not a sensitive indicator of lettuce growth. However, the experimental results of this study revealed that leaf length and height are sensitive indicators of lettuce yield compared to leaf width and the SPAD measurement (Figure 3).

There were linear relationships among the *DW*, leaf length, and shoot height during the 25–50 days following sowing as shown in equations (1) and (2):

*DW* = 0.0194 *L*–0.154 (r^2^ = 0.651) (1)

*DW* = 0.0144 *H*–0.122 (r^2^ = 0.631) (2)

### 3.4. Effects on the Total Nitrogen and Nitrate Concentrations

**Figure 4:**
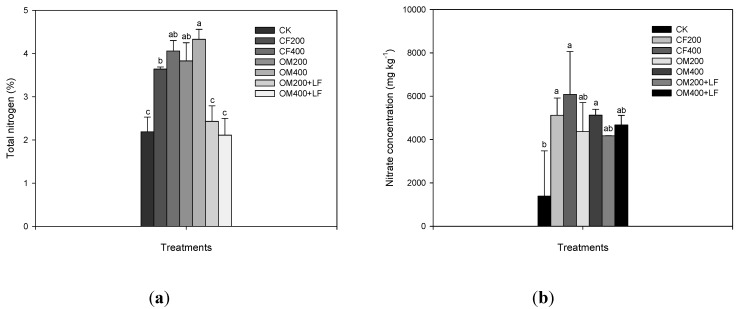
Effects of different nitrogen fertilizer treatments on the (**a**) total nitrogen and (**b**) nitrate concentrations in lettuce after 50 days of cultivation. Values followed by different letters in columns are statistically different (*p* < 0.05).

Relative to the CK fertilizer-treated plants and except the combination of organic fertilizers and liquid fertilizers, the total nitrogen concentrations in the edible parts of the lettuce treated with inorganic and organic fertilizers significantly increased 65–100% (*p* < 0.05; Figure 4a). However, overall, the total nitrogen concentrations increased when fertilizers were applied (Figure 4b).

The edible parts of the lettuce grown in the CK fertilizer had the lowest nitrate concentration (1,391 mg·kg^−1^) while the highest was found in the lettuce treated with inorganic fertilizers. The nitrate concentrations in the lettuce treated with inorganic and organic fertilizers reached 5,000–6,100 and 4,300–5,200 mg·kg^−1^, respectively. Although some of the differences were not significant, applying liquid fertilizers further decreased the nitrate concentration by 4%–10% compared to the lettuce treated with organic fertilizers only. This resulted from the lower nitrate concentrations in the tested soils (Table 1).

### 3.5. Discussion

Like the studies by Chen *et al.* [36] and Petropoulos *et al.* [37], our experimental results revealed that the nitrate accumulation had a close relationship with the amount of fertilizer applied. The edible parts of the lettuce grown in CF200 accumulated 5,120 mg·kg^−1^ of nitrate while 19% more nitrate amassed in the edible parts of the plants treated with CF400 without significant difference. The release of nitrogen in organic fertilizers is slower than that in inorganic fertilizers since organic fertilization typically does not provide nitrogen in a readily accessible form [38]. The lettuce grown in the OM200 and OM200 + LF treatments thus accumulated 14%–19% less nitrate than CF200 although the differences were not significant. This experimental result is in agreement with the results of previous studies [39,40,41] in that the organic fertilizer-amended lettuce accumulated lower nitrate concentrations compared with conventional lettuce. According to earlier studies in Denmark, Estonia, France, and Korea, the mean nitrate content in organic fertilizer-amended lettuce was 1,900 to 2,700 mg·kg^−1^ [42,43,44,45]. 

Lighting conditions influence the nitrate reductase activity and decrease the conversion rate of nitrate to amino acids, leading to a higher concentration of nitrates [9]. Therefore, in agreement with previous studies [46,47] and possibly as a result of the lower artificial lighting conditions compared with natural light, the nitrate content in the lettuce in this experiment was approximately two times higher than that found in the previous literature.

According to the regulatory limits for nitrates in lettuce established by EC Regulation No. 1881/2006, the maximum permissible levels are 4,000–4,500 mg·kg^−1^ during 1 October to 31 March [48], the period during which our pot experiment was conducted. In our experiment, the nitrate content in the lettuce grown in the different treatments was 4,100–5,200 mg·kg^−1^. Only the lettuce grown in OM200 and OM200 + LF complied with the EC regulations since the concentrations reached 4,365 and 4,175 mg·kg^−1^, respectively.

Previous studies indicate that vegetables are the major source of nitrate intake by humans and constitute approximately 40%–92% of the average daily intake [49,50]. Assuming lettuce grown in OM200 and OM200 + LF was the only vegetable consumed, a person eating 400 g of vegetables, as recommended by the World Health Organization, would have dietary exposure to approximately 1,670–1,746 mg of nitrate. For a person weighing 60 kg, consuming this item alone would exceed the U.S. EPA reference dose for nitrate, *i.e.*, 420 mg, by about 3.9 to 4.2 times. According to van Velzen *et al.* [51], the oral bioavailability of nitrate from vegetables is around 100%. Therefore, consuming a balanced diet of vegetables could be an effective method for decreasing the amount of nitrate exposure in humans.

Among the applications in this study, the lettuce treated with inorganic fertilizers showed growth representations similar to those of to the CK-treated lettuce, but also had the highest nitrate concentrations. Compared to inorganic fertilizers, there was better growth when lettuce was grown in organic fertilizer-treated soil. Although there was a 13%–34% decrease in the *DW* compared with organic fertilizer applications only, the additional application of liquid fertilizers increased the estimated *LA* (15%–23%) and decreased the nitrate concentrations (4%–9%). These results show that lettuce grown in soils treated with a combination of organic fertilizers and liquid fertilizers had thinner stems, larger leaves, and lower nitrate concentrations compared with the plants that grew in soil treated with organic fertilizers only. Leaves are the main edible parts of lettuce; in this study, the combination of organic fertilizers and liquid fertilizers in soil, particularly soil treated with OM200 + LF, provided a better lettuce cultivation method.

## 4. Conclusions

Application of nitrogen fertilizers influences the nitrate concentration in the edible parts of lettuce. Our experimental results show that providing lettuce with a combination of organic fertilizers and liquid fertilizers is superior in terms of soil quality, appearance, and the accumulation of nitrate. The inorganic fertilizers and the application of liquid fertilizers frequently increased the electrical conductivity of the soil and thus negatively affected the yield of the growing lettuce. The results of this study provide useful information to farmers and policymakers.
